# Envelope filter sequence to delete blinks and overshoots

**DOI:** 10.1186/s12938-015-0046-0

**Published:** 2015-05-30

**Authors:** Manuel Merino, Isabel María Gómez, Alberto J Molina

**Affiliations:** Department of Electronic Technology, University of Seville, Avd. Reina Mercedes s/n, 41012 Seville, Spain

**Keywords:** Biosignal processing, EOG signal, Envelope filter, Blink, Overshoot

## Abstract

**Background:**

Eye movements have been used in control interfaces and as indicators of somnolence, workload and concentration. Different techniques can be used to detect them: we focus on the electrooculogram (EOG) in which two kinds of interference occur: blinks and overshoots. While they both draw bell-shaped waveforms, blinks are caused by the eyelid, whereas overshoots occur due to target localization error and are placed on saccade. They need to be extracted from the EOG to increase processing effectiveness.

**Methods:**

This paper describes off- and online processing implementations based on lower envelope for removing bell-shaped noise; they are compared with a 300-ms-median filter. Techniques were analyzed using two kinds of EOG data: those modeled from our own design, and real signals. Using a model signal allowed to compare filtered outputs with ideal data, so that it was possible to quantify processing precision to remove noise caused by blinks, overshoots, and general interferences. We analyzed the ability to delete blinks and overshoots, and waveform preservation.

**Results:**

Our technique had a high capacity for reducing interference amplitudes (>97%), even exceeding median filter (MF) results. However, the MF obtained better waveform preservation, with a smaller dependence on fixation width.

**Conclusions:**

The proposed technique is better at deleting blinks and overshoots than the MF in model and real EOG signals.

## Background

The eye’s goal is to project reflected light from an object onto ocular fovea. Eye movements can be grouped into slow and quick [1]. The former make it possible to maintain either a projected image of non-static objects or a projected image when the head is turned (velocity <30°/s), while quick movements prevent an image from being lost as it is projected on the same place in the retina (microsaccadic—steps <0.25°/s), and if there is a quick change of point of view (saccadic—variations <700°/s). The eyelids are the other important element of the human visual system. They moisten, clean and protect eyes from external physical agents. Their movements are called blinks.

Eye and blink movements are used in studies on somnolence, workload, or concentration. Different studies have related eye movements to central nervous system activity [2] and somnolence [3, 4]. In turn, blink rate has been reported as an indicator of sleeplessness and attention/concentration, whereby sleep deprivation raises its frequency and duration, while an increase in attention levels produces a decrease in blinking [5, 6]. Some control interfaces have been based on them: for example, activity recognition or handling a computer through events. Classifying activities are based on pattern detection, as in reading which involves small eye movements from the beginning of a text line and a big shift at the end [7]. Event activities are mainly based on go-and-back movement (GBM) from eyeball center to an extreme: for example, a virtual keyboard [8], a mouse pointer [9], or a wheelchair [10].

**Figure 1:**
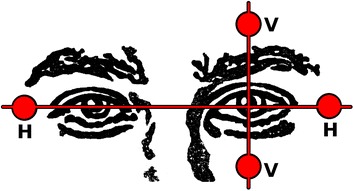
EOG electrode layout. Electrodes H and V record horizontal and vertical eye movements.

Ocular activity can be recorded using several techniques, such as infrared light [11], video camera [12], or electrooculography (EOG), which is our focus. EOG is a well-known eye-tracker technique which measures the electric potential difference between cornea and retina (±1 mV [13]—depending on several factors such as light level [14]) and is recorded when ocular movements occur. It measures electrical activity with Ag/AgCl electrodes placed around the eyes. The most common electrode layout is shown in Figure 1. Two electrodes for each horizontal or vertical direction are employed, providing bipolar data. A monocular configuration is utilized in vertical eye movements, and a binocular setting is used in horizontal shiftings [1]. EOG amplitudes range from 5 to 20 μV/°, so that ±30° ocular movements [15] are quasi linear, and essential frequency components range from 0 to 30 Hz [16]. The duration of a saccade depends on the angle of eye movement, with the most common being under 20°, and lasting from 10 to 100 ms [12]. The time between two consecutive saccades is termed fixation and the average value lies between 100 and 200 ms [17].

**Figure 2:**
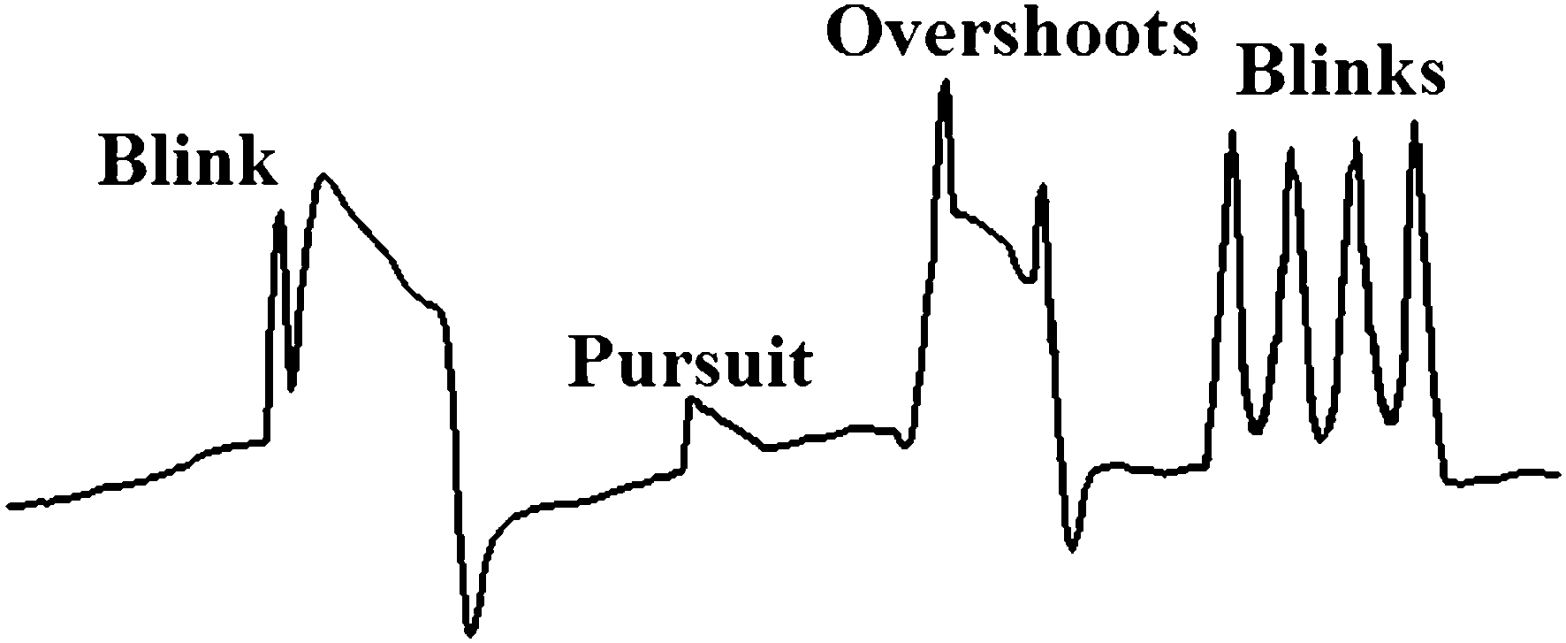
EOG signal. Saccades, blinks, overshoots and pursuit movements.

The eye-tracker system using an EOG faces a series of problems: noise, drift, blinks and overshoots [18]. This paper focuses on the last two. The blinking signal is a bell-shaped noise which overlaps on the electrical activity of eyes, while overshoots are similar to blinking impulses located in the saccadic area and they occur due to target localization error corrected by a secondary saccadic eye movement [19, 20] (Figure 2). Blinks are caused by eyelid movements, while overshoots happen mainly with fast, high amplitude, eye movements. The blink rate of a relaxed individual is between 12 and 19 blinks per minute [21], with an average duration of 100 up to 400 ms [22].

The median filter (MF) is one of the most commonly used techniques for deleting these noises because the saccadic-edge slopes are preserved and noise is attenuated when the duration is at its most buffered [23]. This can be used in online and/or offline processings because it is based on split windows. Two other algorithms based on MF are FIR median hybrid filters (FMH) and weighted FMH filters (WFMH) [24]. The former applies the MF to the output of an FIR filter, whereas in the latter, the MF is obtained for each output of an FIR filter multiplied by a constant. Two versions of WFMH are interesting: center weighted FMH filter (CWFMH) and subfilter weighted FMH filter (SWFMH) [24]. CWFMH leaves all FIR outputs intact, apart from the middle element which is multiplied by a constant. The edges and sinusoidal signals are preserved. SWFMH multiplies the extremes and the central elements remain intact. High frequency noise is removed and the edges are maintained. All these filters were analyzed in Martinez et al. [25]. FMH and WFMH results are not better than MF, while SWFMH produces non-meaningful differences in detection rate in relation to MF. In addition, a serial sequence of MF may improve the results of a single MF [26, 27]. However, a sequence or a single MF encounters several problems for deleting noise [28]. A blink in the saccade vicinity causes the edge slope to be smoothed, whereas a sequence of consecutive blinks produces a squared pulse similar to eye movement (Figure 2). In contrast, small steps are caused by smooth pursuit movements and a big MF mask size delays the saccadic edges.

In this paper, we propose an algorithm for removing overshoots and blinks. The methodology is detailed in the next section, with a description of the proposed filter, and procedures to evaluate processing effectiveness. We then go on to analyze and discuss processing outputs.

## Methods

This section goes into the details of the online and offline versions of the proposed algorithm for processing EOG data (first subsection), and the tests performed to determine effectiveness (second subsection). We performed the tests with our own seven acquired EOG signals to evaluate in real data and simulated EOG signals to measure and verify different processing features. We developed this model because we were unable to find EOG databases with specialist annotations of saccade movements, blinks and overshoots (“Appendices 1, 2”). By using the model we were able to evaluate processing precision versus inserted noise level from blinks, overshoots, and general interferences, and to compare filtered outputs with ideal data. Version 8.0.0.783 of Matlab was utilized to develop and simulate the processings and to analyze their results.

### EOG filter

This section describes the process for deleting blinks and overshoots in the EOG signal. The algorithm is based on obtaining envelopes to signals as occurs in empirical mode decomposition technique [29]; a similar process was used to detect QRS waveforms in electrocardiogram signals [30], to filter peak and spike noise in EEG signals [31], to study foot muscle coordination from EMG data [32], and to obtain power dependencies in neuroimaging data [33]. The next two subsections explain the process for obtaining lower envelope and how it is used to filter; the last two add detail to the proposed technique.

#### Lower envelope

**Figure 3:**
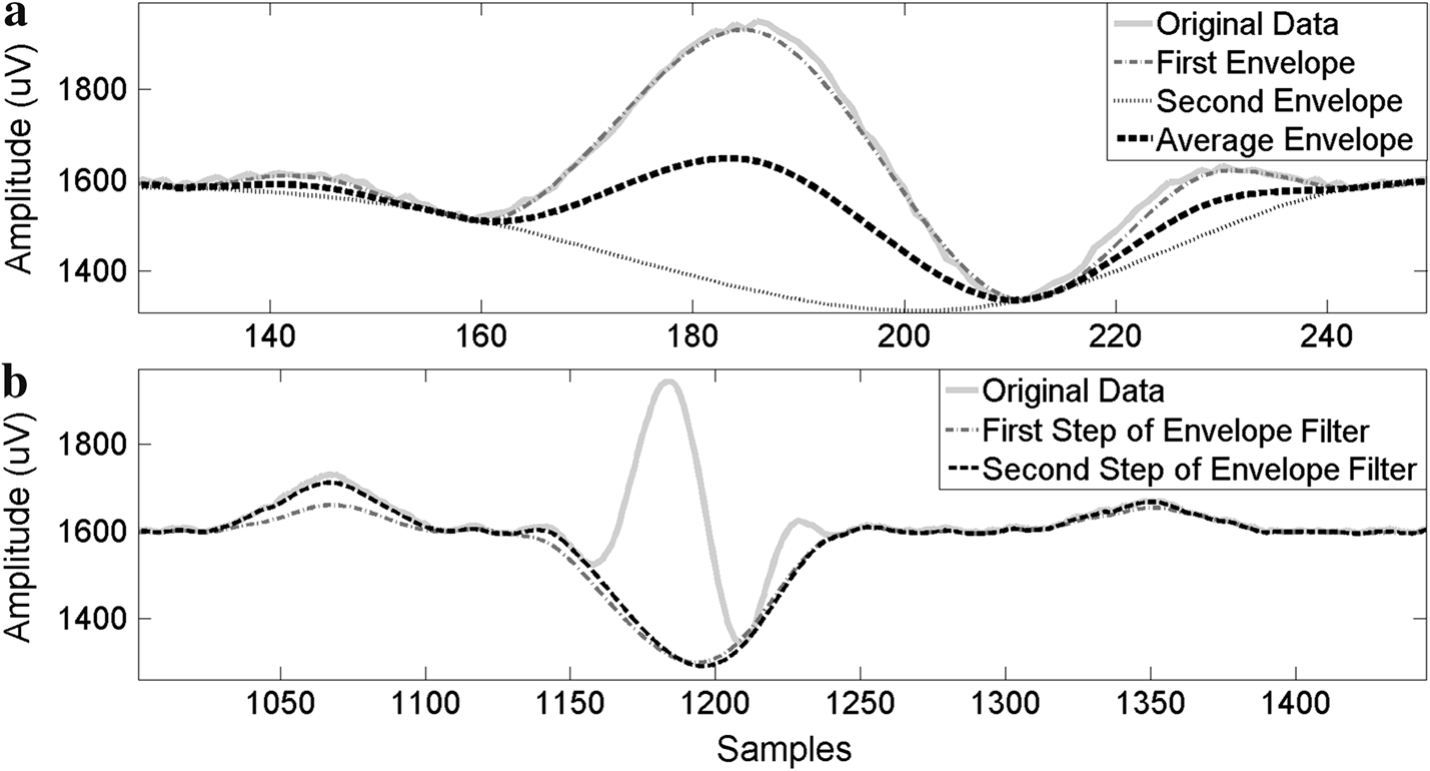
Envelope filter. **a** Steps 1–3 of envelope filter, **b** the result of envelope filter loop.

Essentially, the algorithm finds a set of envelopes of the EOG signal (Figure 3a) by following two steps.

Step 1: Find all local minimums from data.

Step 2: Generate a new signal based on the cubic Hermite interpolant that crosses each extreme and passes by the first and final value of the input data.

This interpolation provides a piecewise cubic function based on values at neighboring grid points using third-degree polynomials with Hermite form, so that a smooth approximation of the EOG signal is obtained.

#### Envelope filter

Let *D* be the input data of size n. Let *F* be a zero vector, also of size n, where the filtered data are stored.

Step 1: Let *E1* be the first lower envelope obtained from *D* by applying the procedure described in previous subsection.

Step 2: Let *E2* be the second lower envelope from applying the procedure to E1 (step 1).

Step 3: Add the average of *E1* and *E2* to *F* (1).

1 $$F = F + \frac{E1 + E2}{2}$$

Step 4: Assign to *D* the difference between data and the previously filtered data (*D* = *D* − *F*). The next iteration is performed on this new *D*.

Step 5: Repeat steps 1–4 twice in total.

The algorithm obtains two lower envelopes (steps 1 and 2). The first of them extracted directly from data and the second based on the first extracted envelope. The first envelope cannot filter blinks or overshoots with a sawtooth-shaped top. For this reason, a second envelope is obtained from the first (Figure 3a). The latter decreases saccade edge slopes excessively. To reduce this negative effect of the second envelope, the average between both envelopes is calculated. Although the blinks and overshoots with sway-shaped tops are not totally filtered, their amplitudes are reduced (Figure 3b). Furthermore, a second iteration causes the output fixations to be closer to input-data fixations.

#### EOG filter: envelope filter sequence (EFS)

**Figure 4:**
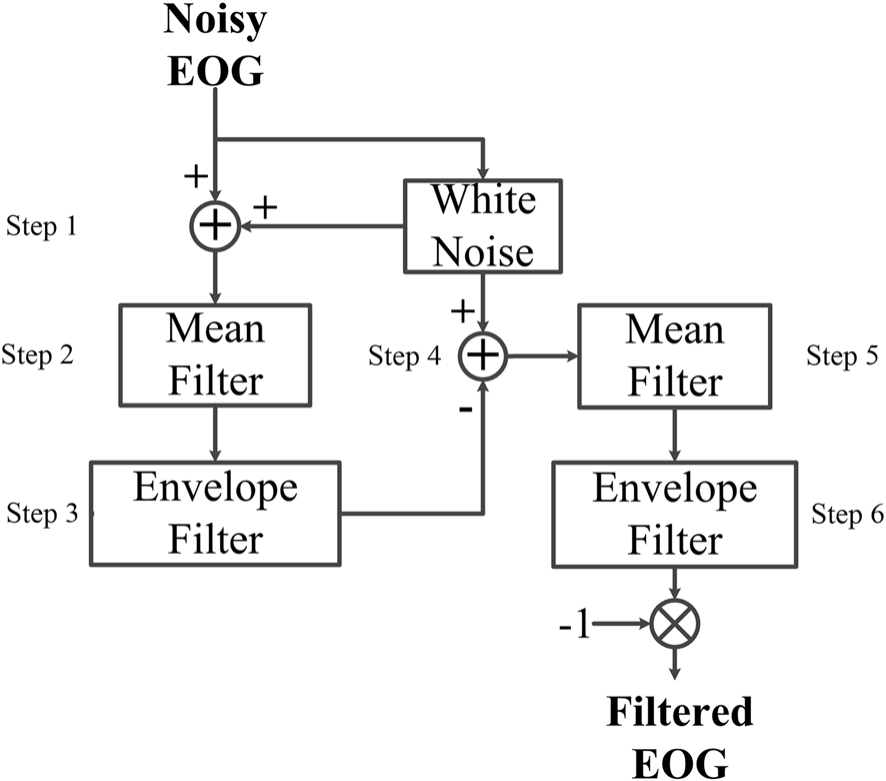
Offline EFS diagram. Steps 1–6.

The procedure to filter blinks and overshoots from EOG, which is shown in Figure 4, is as follows.

Step 1: Generate white noise and add to EOG input. This white-noise signal is known as EFS-WN. It is obtained using (2) where *wn* and *E*_*wn*_ are an initial white-noise signal and its energy, *E*_*s*_ is the energy of input data without DC component and *SNR* is the signal–noise rate measured in decibels of EFS-WN in relation to input data.

2 $$EFS{\text{-}}WN = wn \cdot \sqrt {\frac{{E_{s} }}{{E_{wn} \cdot 10^{\frac{SNR}{10}} }}}$$

Step 2: Apply a mean filter.

Step 3: Employ the envelope filter from the output of step 2.

Step 4: Add the EFS-WN obtained in step 1 to the inverted output of the envelope filter.

Step 5: Apply a mean filter.

Step 6: Employ the envelope filter from the output of step 5. The output must be inverted to keep the original eye-movement directions.

The resulting algorithm is referred to as EFS, and applies two envelope filters (steps 3 and 6). The first deletes concave variations (blinks and top overshoots), whereas the second removes convex curves (down overshoots). EFS-WN is added to cause oscillations in the data (steps 1 and 4). The envelopes are more similar to original data when small variations happen, therefore EFS-WN amplitude has to be small. The output of step 3 is inverted to delete down overshoots correctly (step 4). The mean filters are used to delete small sway-shapes on the top of blinks and overshoots (steps 2 and 5). Thus, sawtooth shapes are generated through the EFS-WN and mean filters. The output of the algorithm must be inverted to maintain initial ocular-movement direction.

Envelope filter sequence supposes that blinks have concave shapes (Figure 2). For this reason, lower envelope is its core. Nevertheless, there are schemes where blink form is convex. In such cases, the input data must be inverted before applying this processing.

#### EFS: online version

**Figure 5:**
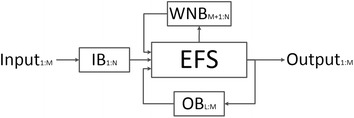
Online EFS diagram. [1:N]: Input buffer length; [1:M]: input/output data length (M < N); [M + 1:N]: overlapping width; [L:M]: fragment size of filtered data (L ≤ M).

Some systems, for example control interfaces, require real-time processing to achieve their goals. An online version is described in this subsection and Figure 5.

Step 1: Three persistent FIFO (First In, First Out) buffers must be established: input-data buffer (IB), white-noise buffer (WNB), and another containing the final fragment of EFS output (OB). The aim of the EFS is to filter blinks and overshoots, so IB length must be wide enough to contain at least one blink. This buffer acts as a sliding window with overlapping. The aim of the overlap is to prevent that part of output becoming deformed because blink waveform is not stored completely in IB. WNB contains the white noise of overlapped data from the previous window. This stops local minimums from this fragment changing in the next execution, and output showing gaps. Finally, output derivability is a desired feature, thus OB is used as a link between iterations and envelopes must cross for their data.

Step 2: When IB is full, calculate EFS-WN from EOG input, join with WNB, and add to IB.

Step 3: Apply a mean filter.

Step 4: Employ the envelope filter, so that each envelope beginning must cross for OB. Two considerations have to be taken into account. First, step 2 of the “Lower envelope” changes slightly. It says that the interpolation must cross the first sample of the input signal, but now, this is replaced by OB. Second, the envelope filter makes two interactions, and OB is used in the first. In the second, OB is replaced by a new zero vector.

Step 5: Add EFS-WN to the inverted output of step 4.

Step 6: Apply a mean filter.

Step 7: Repeat step 4. Invert its output.

Step 8: Store the overlapping segment of EFS-WN in WNB and the final fragment of output in OB.

Step 9: Returned filtered data are not overlapped. Bell-shaped partial waveforms stored in IB may reduce the effectiveness of filtering. To prevent this effect, processing output discards the overlapped buffer segment.

### Procedure

Nine tests were conducted. The first three determined the EFS parameters for offline and online versions. The other tests compared filtering results of EFS and MF. According to [25], MF length was set to 300 ms. The first eight tests used 100 signals from the model described in “Appendix 1”. Henceforth, the model name is EOG system generator (EOG-SG). The main features of these signals are explained in this subsection. However, more details are given in “Appendix 2”. The ninth test compared MF and both versions of EFS from real EOG signals. All tests applied a 30-Hz-lowpass filter [16].

Envelope filter sequence parameters were established in Tests 1 and 2: they were EFS-WN amplitude and mean-filter length. EFS parameters were calculated versus sampling rate. In both tests, one of the EFS parameters was set and the other changed. Test 1 obtained EFS-WN amplitude by increasing *SNR* by 1 dB from 1 to 60 dB in (2) for the same input data. Energy of the input signal was used as *E*_*s*_, and mean-filter length was set at 31.25 ms. With the best EFS-WN amplitudes set in Test 1, mean-filter length was modified from 2 to 100 samples. The best values of EFS parameters were obtained through correlation coefficients (CC) based on Agrawal and Gupta [34] whose expression was (3), where *L* was the number of samples, *EM* was the ideal signal without overshoots and blinks obtained from (17) in “Appendix 1”, and *NI* was the non-ideal signal, so that it could have been filter output or input data. In this case, *NI* was the output data. This feature measured the similarity between signals and its value ranged between 0 and 1. The selected EFS parameters had a CC value over 0.97.

3 $$CC = \frac{{\left( {\sum\nolimits_{i = 1}^{L} {EM_{i} \cdot NI_{i} } } \right)}}{{\left( {\sum\nolimits_{i = 1}^{L} {\left( {EM_{i} } \right)^{2} } } \right) \cdot \left( {\sum\nolimits_{i = 1}^{L} {\left( {NI_{i} } \right)^{2} } } \right)}}$$

Test 3 determined the lengths of three buffers and the overlapping area of the online version of EFS. The values of parameters obtained in Tests 1 and 2 were set. Buffer lengths were modified in each iteration, such that IB increased by 0.1 s in each iteration from 0.6 up to 1 s; overlapping area and WNB width changed from 0.1 to 0.4 s, increasing 0.1 s (WNB depends on overlapping); and OB was modified between 0.05, 0.10 and 0.15 s. The CC and root mean square error (RMSE) (4) were obtained in each case.

4 $$RMSE = \sqrt {\frac{{\sum\nolimits_{i = 1}^{L} {(EM_{i} - NI_{i} )^{2} } }}{L}}$$

Tests 1–3 used EOG-SG signals with 100 GBMs, blinks and overshoots. Different features of the model were set randomly. The range of eye movement oscillated between −40° and +40°, with horizontal fixations whose time was established between 0.6 and 1.5 s. Blink width varied between 0.3 and 0.55 s with a frequency of 19 blinks per minute. They happened in periods between GBMs which this time was set between 3 and 5 s. In addition, sampling rates in Tests 1 and 2 were {128, 256, 360, 512, 1,200, 2,400} Hz, while Test 3 used 128 Hz, because it was considered that the results of the first two tests were similar for all sampling rates.

With EFS parameters set, Tests 4–8 analyzed the ability of MF and EFS filters to delete blinks and overshoots and waveform preservation for a 128 Hz sampling rate. This value was selected to reduce model and analysis computing time.

Test 4 measured the level of bell-shaped removal through blink signals without saccadic movements. Blink durations were increased 0.1 s in each iteration from an initial interval of [0.1, 0.2] s up to [0.4, 0.5] s. Blink frequency varied randomly between 12 and 22 blinks per minute. Besides CC and RMSE from filter output, we calculated the percentage decrease of blink amplitudes and percentage of processed blinks whose output amplitudes remained higher than the 25% raw blink amplitude. Tests 5–7 studied waveform preservation of both filters versus fixation time without blinks and overshoots. The duration varied randomly from an initial interval between 0.3 and 0.4 s up to fixations between 1.4 and 1.5 s. The interval width was increased by 0.1 s in each new signal. Test 5 focused on signals with 1,000 GBMs; Test 6 used 125 stair-shaped waveforms in each signal to simulate reading activity; and Test 7 analyzed signals with 1,000 consecutive random saccadic movements to imitate natural eye movements. In Test 6, the ocular shiftings in a stair waveform moved from the start point of −15° up to the final position of +15° where the number of saccades oscillated randomly between 6 and 10. Tests 5 and 7 used saccades whose values were between −40° and +40°. In these tests, the CC and RMSE were calculated from filtered data. Test 8 analyzed the effect of fixation slopes in 1,000 GBMs signals with overshoots and blinks, as defined for Tests 1–3, but with a sampling rate of only 128 Hz. The difference with respect to these tests was the fixation slope, that is, the variation of amplitude between two consecutive saccades (“Appendix 1”—second subsection). This dropped during fixation time, with the decrease ranging from 5% in each iteration from an initial interval [0, 5]% up to [35, 40]%. Note that variation in the fixation slopes made it difficult to restore their horizontal-shaped waveforms. Hence, this test established the signal with variations in the fixation slope as the ideal signal, as defined in (17). This test calculated the following features: CC, RMSE, SNR, saccadic slope variations (SSV) and the number of non-filtered overshoots from the filtered signal. They were obtained for input and output signals. SNR was obtained by (5) where *NI* could be the input signal or the output data; thus, it measured how they were affected by noise. SSV was calculated by (6) where *P* was the number of saccades, *S*(*i*) was the set of samples of the saccade slope, and *FO* was filter output.

5 $$SNR = 10\log_{10} \left( {\frac{{\sum\nolimits_{i = 1}^{L} {(EM_{i} )^{2} } }}{{\sum\nolimits_{i = 1}^{L} {(EM_{i} - NI_{i} )^{2} } }}} \right)$$

6 $$SSV = \frac{100}{P} \cdot \sum\limits_{i = 1}^{P} {\left( {1 - \frac{{\sum\nolimits_{j = 1}^{S(i)} {\left( {FO_{j} - FO_{j - 1} } \right)} }}{{\sum\nolimits_{j = 1}^{S(i)} {\left( {EM_{j} - EM_{j - 1} } \right)} }}} \right)}$$

The ethics committee of the University of Seville accepted Test 9 with real data from individuals. This one was performed with 55 min of real EOG data from seven signals from three subjects: eye movements were recorded while the individual watched a film (natural eye movement), read a book (stair-waveform), did GBM, and watched dark screen for null activity (blinkings). Only the vertical channel was obtained for natural movements, whereas horizontal and vertical shiftings were recorded for other signals. They were recorded using the bioamplifier model gtec gUSBamp, and version 2.0 of the BCI2000 software [35]. Their features are summarized in Table 1. Sampling rate was 1,200 Hz for natural movement, and 256 Hz for the others. Notch to remove power line interference and 30 Hz lowpass filters were applied to all of them [16]. The reason for these sampling rates was that the data we used were recorded before the EFS technique and this paper’s tests were developed. Data were filtered through MF and offline and online versions of EFS. Data were then split to select only intervals of blinks, saccades and overshoots. Segments of raw data and processing outputs were normalized between 0 and 1. Normalization values of raw data were applied to filtered signals. The extracted features were: mean of SSV for saccade slopes; mean of overshoot amplitude reduction; and summation of blink area reduction.

**Table 1:** Real EOG signal features

Signal	Time (min)	Sampling rate (Hz)	Blinks		Saccades			Overshoots	
			Number	Width (ms)	Number	Fixation width (ms)	Slope width (ms)	Number	Width (ms)
Blinks	8	256	132	479 ± 8	0	0	0	0	0
GBM	4	256	19	496 ± 22	73	1,637 ± 42	155 ± 8	25	154 ± 10
Natural	21	1,200	103	268 ± 13	278	2,365 ± 165	156 ± 6	49	113 ± 6
Reading	22	256	28	287 ± 12	1,612	301 ± 4	44 ± 0.48	16	116 ± 11

Real EOG features. Mean and standard error are shown for blink, saccade, and overshoot times for each real signal.

Outlier values of all measured features were avoided using the interquartile-range method (7), where *f* were values of the feature used, and *Q1* and *Q3* were quartiles 1 and 3 respectively.

7 $$f \in [{Q1 - 1.5(Q3 - Q1),} \quad {Q3 + 1.5(Q3 - Q1)]}$$

## Results

**Figure 6:**
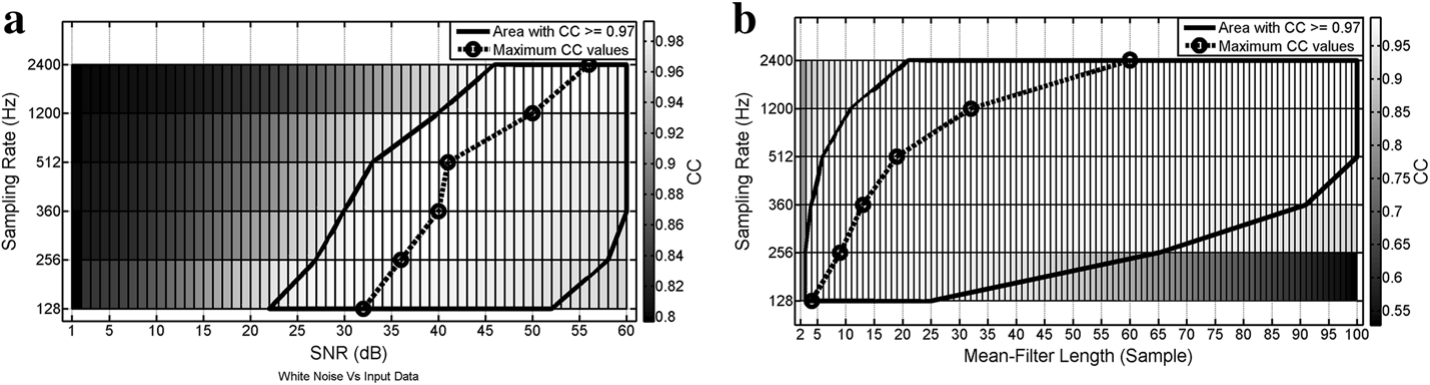
Results of Tests 1 and 2. **a** Best amplitudes of FS-WN; **b** best lengths of mean filter.

The analysis of results of Tests 1 and 2 provides EFS parameters for its offline version. They are summarized in Table 2 and Figure 6. The selected intervals have a CC over 0.97. The SNR of EFS-WN and mean-filter length increased with sampling rate. These SNR values translated into smaller EFS-WN amplitudes, because more noise data were recorded. Furthermore, when mean-filter length time was calculated, it appeared as more stable.

**Table 2:** Best values of EFS parameters

Sampling rate (Hz)	Test 1		Test 2	
	SNR (dB)	Best value (dB)	Mean-filter length (ms)	Best value (ms)
128	[22, 52]	32	[15.63, 187.5]	31.25
256	[27, 58]	36	[7.81, 250.00]	35.16
360	[30, 60]	40	[8.33, 250.00]	36.11
512	[33, 60]	41	[9.77, 193.36]	37.11
1,200	[40, 60]	50	[8.330, 82.50]	26.67
2,400	[46, 60]	56	[8.330, 41.25]	25.00

Best values of EFS parameters versus sampling rate. CC values are 0.97 higher in each interval of the table.

**Figure 7:**
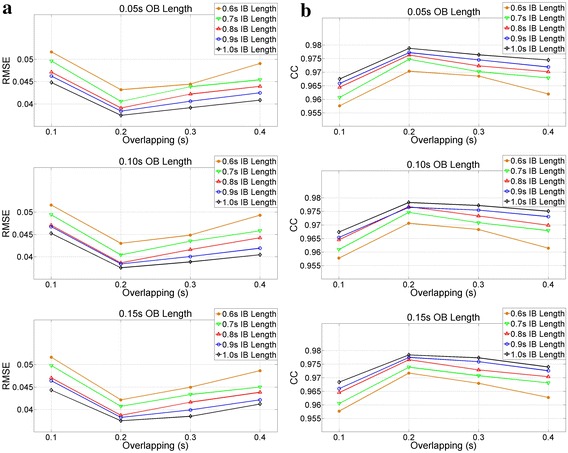
Results of Test 3. Matching IB length of {0.6, 0.7, 0.8, 0.9, 1.0} s with OB length of {0.05, 0.10, 0.15} s with overlapping of {0.1, 0.2, 0.3, 0.4} s. **a** Root mean square error; **b** cross correlation.

Test-3 results provided buffer lengths for the EFS online version (Figure 7). There was practically no change in error in relation to OB. The smallest length was selected (0.05 s). In addition, an overlapping area of 0.2 s produced the best results for all cases. Finally, output data were more similar to the ideal signal when IB width enlarged, as more signal information was stored. However, a greater length produced an increased delay in real time systems. Thus, an IB of 0.7 s was selected, because this length obtained a CC over 0.97. The online EFS parameters, that is, EFS-WN amplitude and mean-filter length, were equal to the offline version, because we supposed that each segment of signal had an SNR value similar to the total signal. In contrast, Test 3 CC results were similar to Tests 1 and 2, thus the results of Tests 4–8 can be extrapolated to the online version.

**Figure 8:**
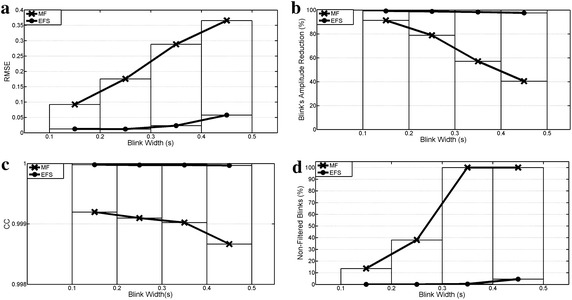
Blink removal. **a** Root mean square error; **b** percentage decrease of blink amplitudes; **c** cross correlation; **d** percentage of blinks whose amplitudes after filtered processing exceeded 25% of original amplitudes.

Test-4 results are shown in Figure 8. The median filter’s ability to remove bell-shaped interference decreased with blink duration, while it remained constant with the EFS filter (the error was between 6 and 15 times smaller). In this way, blink amplitude was reduced from 99 to 97.5% with the EFS algorithm in all cases, whereas MF effectiveness fell abruptly from 91 to 40% as bell-shaped width increased. In addition, the number of blinks whose amplitude exceeded 25% of the original value increased as the duration increased, such that it was only 4.6% for EFS processing in the worst case, and always far below the MF.

**Figure 9:**
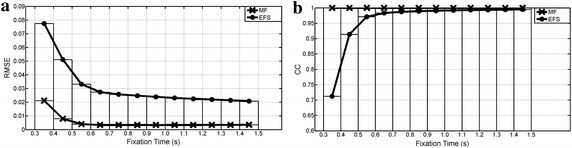
Preservation of GBM waveform. Pulse width changes from interval [0.3, 0.4] s up to [1.4, 1.5] s. **a** Root mean square error; **b** cross correlation.

**Figure 10:**
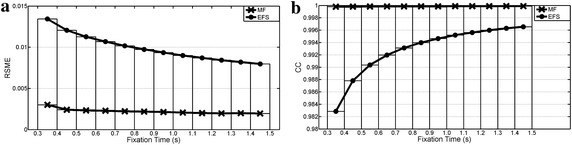
Preservation of stair waveform (reading activity). Step width changes from interval [0.3, 0.4] s up to [1.4, 1.5] s. **a** Root mean square error; **b** cross correlation.

**Figure 11:**
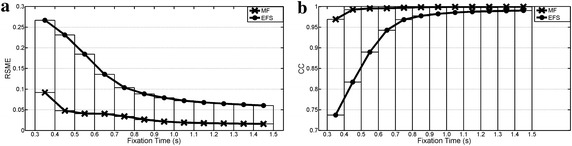
Preservation of random waveform. Step widths change from interval [0.3, 0.4] s up to [1.4, 1.5] s. **a** Root mean square error; **b** cross correlation.

Waveform preservation test results (Tests 5–7) are summarized in Figures 9, 10 and 11. RSME shows that MF fitted better than EFS in all cases. These differences decreased as fixation duration increased. Both processings had a much larger error with random ocular movements versus other cases, while the reading activity produced the best error values. EFS showed CC values over 0.97 when fixation widths were higher than 0.5 and 0.7 s for GBM and random ocular movements, whereas its smallest value was 0.98 for reading the EOG signal. Meanwhile, the MF had no fixation problems, with the output signal being almost equal to the input signal (CC > 0.99), when input was free of blinks and overshoots.

**Figure 12:**
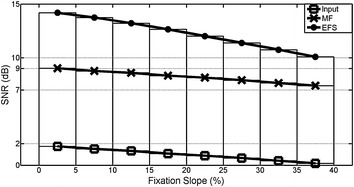
Fixation slope effects. Variation of SNR of input data, MF and EFS outputs. Slope changes from interval [0, 5]% up to [35, 40]%.

Results of Test 8 are summarized in Table 3 and Figure 12. SNR shows that the capacity to obtain the ideal signal with MF and EFS decreased with the fixation slope. EFS processing was more effective at restoring the ideal signal as shown in all table features. However, the fixation-slope effect produced smaller variations of SNR, CC and RSME in MF.

**Table 3:** Fixation slope effect

	Fixation slope (%)							
	[0, 5]	[5, 10]	[10, 15]	[15, 20]	[20, 25]	[25, 30]	[30, 35]	[35, 40]
Input								
SNR (dB)	1.754 ± 0.014	1.509 ± 0.015	1.330 ± 0.014	1.079 ± 0.014	0.887 ± 0.013	0.664 ± 0.016	0.41 ± 0.011	0.181 ± 0.014
CC	0.805 ± 4e−4	0.798 ± 5e−4	0.793 ± 5e−4	0.785 ± 5e−4	0.779 ± 4e−4	0.771 ± 6e−4	0.763 ± 3e−4	0.755 ± 5e−4
RMSE	0.145 ± 1e−4	0.146 ± 1e−4	0.145 ± 1e−4	0.145 ± 8e−5	0.146 ± 1e−4	0.146 ± 1e−4	0.146 ± 1e−4	0.146 ± 1e−4
EFS								
SNR (dB)	14.188 ± 0.021	13.742 ± 0.022	13.216 ± 0.021	12.640 ± 0.023	12.017 ± 0.025	11.380 ± 0.028	10.750 ± 0.026	10.080 ± 0.025
CC	0.982 ± 8e−5	0.980 ± 11e−4	0.978 ± 12e−5	0.974 ± 15e−5	0.970 ± 20e−5	0.965 ± 25e−5	0.959 ± 30e−5	0.952 ± 33e−5
RMSE	0.035 ± 1e−4	0.036 ± 1e−4	0.037 ± 1e−4	0.038 ± 1e−4	0.40 ± 1e−4	0.042 ± 1e−4	0.044 ± 1e−4	0.047 ± 1e−4
SSV (%)	50.02 ± 0.026	50.17 ± 0.026	50.28 ± 0.026	50.28 ± 0.027	50.22 ± 0.027	50.21 ± 0.029	50.09 ± 0.030	50.09 ± 0.031
MF								
SNR (dB)	9.020 ± 0.019	8.768 ± 0.020	8.597 ± 0.019	8.335 ± 0.019	8.147 ± 0.015	7.914 ± 0.021	7.650 ± 0.017	7.407 ± 0.020
CC	0.944 ± 22e−5	0.941 ± 25e−5	0.939 ± 25e−5	0.936 ± 25e−5	0.933 ± 21e−5	0.930 ± 31e−5	0.926 ± 24e−5	0.922 ± 32e−5
RMSE	0.041 ± 9e−5	0.041 ± 8e−5	0.041 ± 8e−5	0.042 ± 7e−5	0.042 ± 7e−5	0.042 ± 8e−4	0.042 ± 8e−5	0.042 ± 8e−5
SSV (%)	1.27 ± 5e−3	1.305 ± 6e−3	1.376 ± 6e−3	1.456 ± 6e−3	1.484 ± 6e−3	1.617 ± 7e−3	1.803 ± 7e−3	1.971 ± 8e−3

Analysis of fixation slope effects (mean ± standard error). SNR, CC and RMSE were calculated using the ideal signal as reference.

The best values of SNR, CC and RMSE were reached when fixation slopes were between 0 and 5%. EFS processing obtained a good SNR value for all cases (>10 dB), CC increased about 22% and RMSE dropped to 76% in relation to input data, whereas the MF SNR value was below 10 dB, CC increased around 17% and RMSE dropped to 72%.

While saccade slopes in all cases remained virtually unaltered in MF, in EFS, they dropped to 50%. Both techniques filter all overshoots completely (100% deleted).

**Figure 13:**
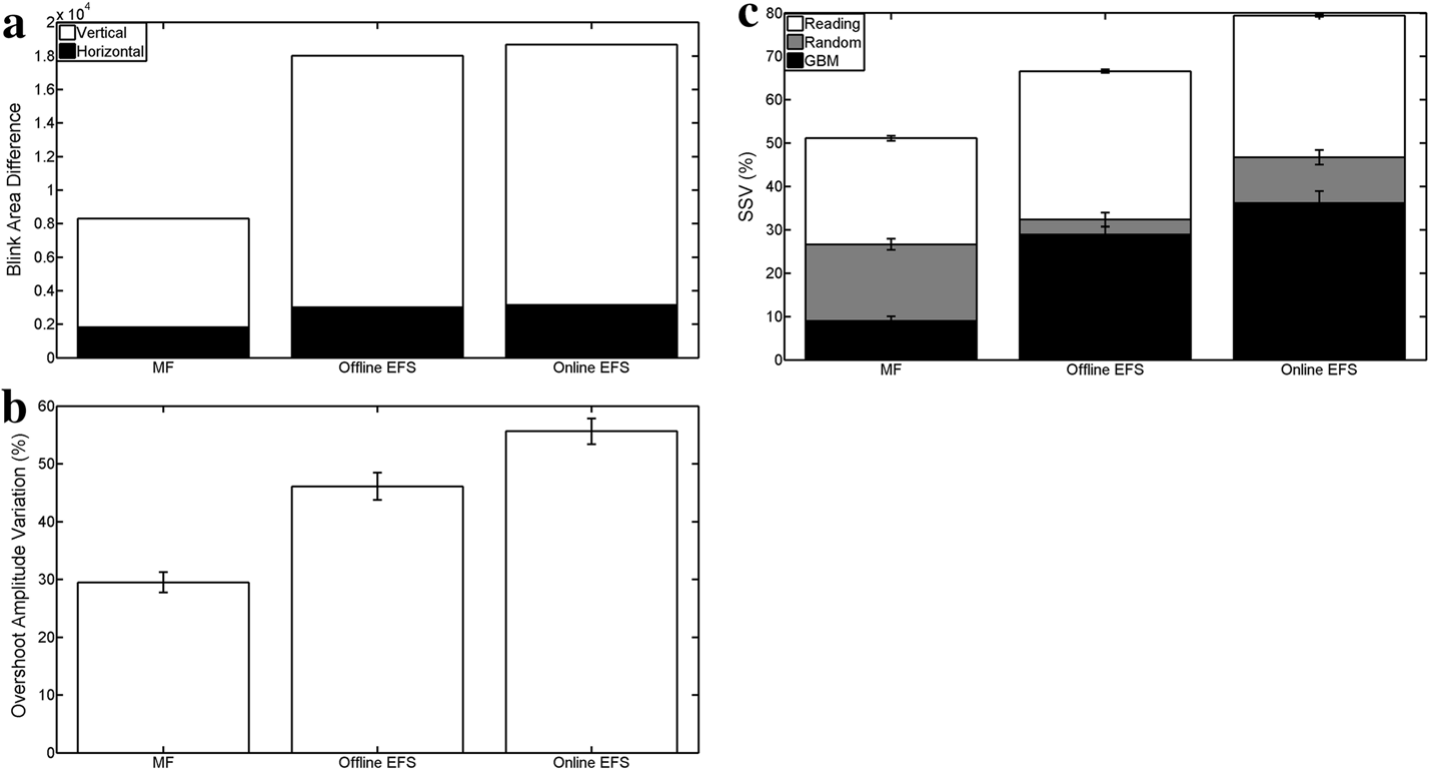
Result of real EOG processing. Results of MF, offline and online EFS. **a** Summation of blink area reduction; **b** overshoot amplitude variation; **c** saccade slope preservation for reading, natural and go-back eye movements.

**Figure 14:**
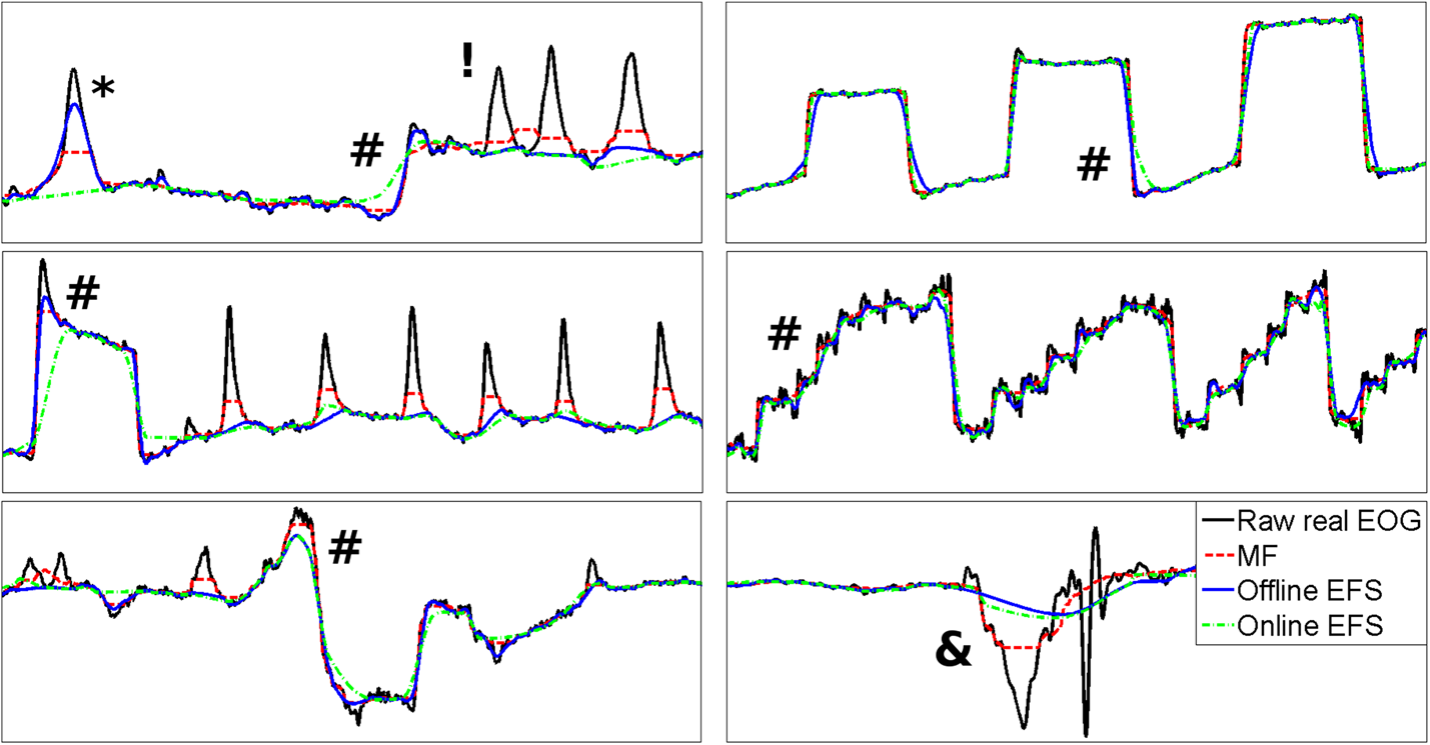
Test of real EOG. Visual analysis of results of MF, offline and online EFS.

Finally, Figure 13 shows the features measured in Test 9 for real EOG signals, with a visual analysis in Figure 14. EFS parameters were set from Table 2 for sampling rates of 256 and 1,200 Hz. However, online EFS-WN SNR was set to 27 and 30 dB for better results. A 300 ms MF was much less capable of deleting blinks, with the decreased area being 53.96 and 55.61% lower than the offline and online EFS versions (Figure 13a). Blink interference in horizontal movements was also 39.58 and 42.45% lower. However, non totally-filtered blinks retained a greater amplitude with EFS (Figure 14, markers *). When there was a short time between two blinks, MF generated an artificial pulse between them (Figure 14, marker !), and this never occurred with EFS. Overshoot amplitudes only dropped around 30% for MF (Figure 13b), while EFS versions obtained better results (46 and 55%). However, saccadic slopes were better in MF (Figures 13c, 14, markers #). For reading activity, MF preserved 49% of slopes, whereas EFS retained 34 and 20% for offline and online versions. All processings retained better for other signals: 74, 68 and 54% of natural-movement slopes were conserved, and 91, 72 and 64% of GBM slopes were preserved. Finally, spike perturbations were filtered in both cases (Figure 14, marker &), but EFS reduced them to a greater extent.

## Discussion

Bell-shaped waveforms, such as blinks and overshoots, are unwanted elements in some control interfaces or activity classifiers [7–10, 18], because they reduce their effectiveness. The offline and online versions of the technique proposed in this paper achieved high and stable levels of elimination with low response delays (EFS-WN, mean filter, finding local minimums and interpolation are linear operations), with their amplitudes decreasing by more than 97%, which was far better than the 300 ms-length MF. This fact was confirmed with real EOG data. Thus, control systems, like the complex state machine developed in Merino et al. [18], could clearly be simplified by using EFS preprocessing. In turn, the ability of EFS to detect bell-shaped waveforms could be used to send commands when a voluntary blink happens.

Some systems are based on GBM eye movements as commands, whereas others use a saccade-movement sequence to determine which activity has occurred. In these systems, EFS processing may not be useful with short fixations, and a fixation-time threshold is required to achieve a satisfactory level of waveform preservation, for example GBM must exceed 0.5 s. The reason for this is that there are few or no local minimums with small fixations. The envelope filter uses these minimums to delete bell-shaped interferences and to be closer to input data through an average from two envelopes. Too few minimums may mean that the average is not similar to input fixation, because the first lower envelope may be free of local minimums, and the second is obtained from those values. Hence, the distance between the envelopes may be considerable. This may be made worse if an input fixation interval does not have local minimums. In this case, there is no similarity between the two envelopes.

Envelope filter sequence requires small oscillations of input data to reach a high level of similarity with ideal EOG signals. The objective of EFS-WN is to increase the number of local minimums. However, blinks and overshoots are not removed when the top or its peaks are sawtooth shaped. For this reason, to reduce sway form, a mean filter is applied after adding EFS-WN. High EFS-WN amplitudes may introduce large swayings on bell-shaped peaks, rendering the mean filter ineffective, and a long mean filter may excessively reduce the number of local minimums. Thus, EFS-WN for the offline EFS version is set at a low value (32 dB) for 128 Hz, and this decreases with a larger sampling rate, because more oscillations (noise) are recorded and EFS-WN may be smaller, or even unnecessary. For the online version, EFS-WN amplitudes are greater (27 versus 36 dB for 256 Hz for offline) due to lower window energy. Meanwhile, the mean filter smoothes out data and deletes the sawtooth top of blinks and overshoots. Therefore its length, between 25 and 32 ms, is more constant and independent of the sampling rate.

Applying a highpass filter to the EOG signal causes the fixation slopes to drop (Table 3). This may mask a subset of small variations inserted by EFS-WN. Few local minimums are found and envelopes may not be close enough to fixation periods, thereby reducing similarity, as shown in Test 5. Increasing these slopes causes SNR input to drop. Nevertheless, this fact is inverted with the filtered processings, with a good SNR level and high similarity being reached. Thus, EFS should be applied before the highpass filter to avoid this fixation-slope effect.

An important difference with the techniques based on MF is that a shifting window is not required, so EFS may be applied to all input data. The effectiveness of MF depends directly on window length. A low value may not totally remove blinks or overshoots, while a large width may modify saccadic positions. However, MF is relatively robust to sawtooth-shaped tops, waveform preservation is high (higher than EFS), and it obtains a good SNR value for decreasing fixation slopes (lower than EFS). However, it has difficulty deleting blink places in the saccade neighborhood and consecutive blinks. EFS versions remove the problems of neighborhood and sequence, fixation period error is reduced, and saccade positions are maintained. Furthermore, real data confirm that MF retains a better saccade slope. This fact may be very important for an activity classifier, but it may have less impact in control systems.

## Conclusion

The EFS algorithm for filtering EOG data has demonstrated that it is highly capable of removing bell-shaped waveform noise without changing saccade positions: blink amplitudes decreased by 97%, and only 5% of them maintained a value over 25% of the initial value, and overshoots were considerably reduced. However, saccadic slopes were smoothed and we found a limit of fixation duration. In contrast, MF was less capable of reducing amplitudes of this kind of interference, but was better at maintaining slopes, with a smaller dependence on fixation width being obtained.

The paper described an online implementation of EFS with a similar level of effectiveness to the offline version. Hence, the EFS algorithm can be used by a control interface based on EOG signals to manage devices such as PCs, activity classifiers, and/or affective computing systems.

## Figures and Tables

**Figure 15:**
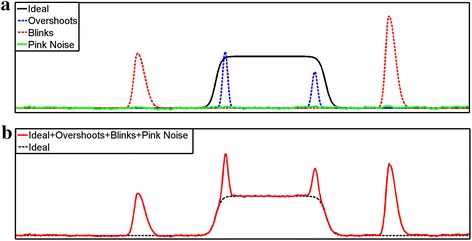
Model EOG signal from EOG-SG. **a** Individual elements; **b** joining of all components.

**Figure 16:**
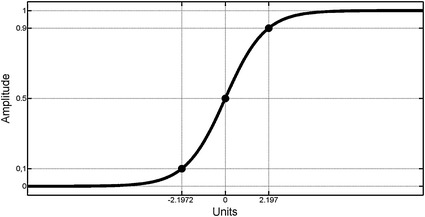
Saccade model. Sigmoid function with *a* = 0 and *b* = 1.

**Figure 17:**
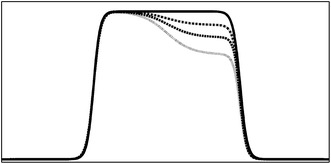
Different fixation slopes. Model EOG signal with four different fixation slopes.

## Appendix 1: EOG system generator

A model of the EOG signal is described in this appendix. This one can measure and verify different EOG features. This model was developed because we were unable to find databases with specialist annotations of saccade movements, blinks and overshoots. The model allows us to evaluate processing precision versus inserted noise level from blinks, overshoots, and general interferences, and compare filtered outputs with ideal data.

An EOG signal may be split into (8)

8 $$EOG(t) = EM(t) + O(t) + B(t) + n(t)$$

where EM(t) is the ideal signal consisting of eye movements, O(t) contains overshoots, B(t) are blinks, and n(t) is additive noise (Figure 15). These functions are described in the following subsections. The model of saccades and fixations is explained in first three subsections, it is generalized in the fourth subsection, where EM(t) is obtained. Overshoot and blink models are described in last two subsections.

### Saccades model

Saccade movements cause a rapid signal variation, whereas fixations maintain the electric level from saccades [1, 17]. They can both be modeled with a sigmoid function (9). This is defined for all real numbers, its rank is (0, 1), with two horizontal asymptotes in 0 and 1 (Figure 16). Parameter *a* shifts it along the abscissa axis, while parameter *b* contracts/dilates it, so that the slope changes with this value.

9 $$S\left( x \right) = \frac{1}{{1 + e^{ - x} }}\mathop{\longrightarrow}^{{{x\,=\, \frac{t - a}{b}}}}S\left( {\frac{t - a}{b}} \right) = \frac{1}{{1 + e^{{ - \frac{t - a}{b}}} }}$$

The main variations of the sigmoid function are found in the interval $[ {a - b{ \ln }\left( { 1/0. 1{-} 1} \right),\quad a - b{ \ln }\left( { 1/0. 9{-} 1} \right)}]$, where it ranges from 0.1 up to 0.9, which is 80% of its slope. Parameter *b* controls the interval amplitude, so it can be used as a model of saccadic shiftings. Thus, supposing the linear relation between eye-movement angles and saccade time widths, with the average time of EOG-saccades slope known as 100 ms for a 20° movement [12], then *b* is defined as (10)

10 $$b(A,\rho ) = A\rho \left( {\frac{0.9}{2\pi \ln (9)}} \right)$$

where 0 ≤ *A* < π is movement angle in radians, 0 < *ρ* < +∞ is a random factor of variability of saccades (RFVS) to involve variations in the slopes.

### Fixations model

Ocular position is maintained after each saccadic movement [17]. This is termed fixation. Under ideal conditions, the electrical level reached is upheld and only changes when a new saccade occurs. In contrast, applying a high pass filter to the EOG signal is a normal action because this shows a DC level. This filter causes the fixation slope to decrease, because the signal is constant in them. So, Eq. (11) models this behavior, where *δ* and *γ* are start time and duration of decrease, and *m* is decreased level, whose value is between 0 and 1. Note, 98% of the slope is in interval [0, 1] when *δ* = 0 and *γ* = 1.

11 $$D\left( {m,t,\delta ,\gamma } \right) = 1 - mS\left( {\ln (99) \cdot \left( {\frac{2(t - \delta )}{\gamma } - 1} \right)} \right)$$

A high pass filter could be used instead of (11), but fixation slope control is lost. So, (11) through parameter *m* handles this EOG feature better.

### Saccade-fixation model

A fixation always happens after a saccade [1, 17]. So, this relation is drawn in Figure 17 and is defined in Eq. (12)

12 $$SF\left( {t,a,b,m,\delta ,\gamma } \right) = S\left( {\frac{t - a}{b}} \right) \cdot D(m,t,\delta ,\gamma )$$

where

13 $$\delta = a - b\ln \left( {\frac{1}{0.99} - 1} \right) > a - b\ln \left( {\frac{1}{0.9} - 1} \right)$$

is the start time of the decreased fixation slope and is located after the saccade and coincides with the time instant when (9) is 0.99. Furthermore, the fixation slope time depends on when the next saccade occurs. If *a*_*2*_ and *b*_*2*_ are the next saccade localization and its slope, then fixation width is defined as

14 $$\gamma = a_{2} - b_{2} \ln \left( {\frac{1}{0.01} - 1} \right) - \delta$$

such that the end of fixation coincides with the time instant when (9) is 0.01 for the next saccade (Figure 17).

### Saccade sequences

Eyes are constantly in movement, so it is possible to establish a movement sequence as a set of saccades and fixations whose initial and final positions are the center of the eyeball. A sequence of saccades is established as a family of curves in (15), returning to the center position as (16)

15 $$\begin{aligned} \hfill SS(t,A,a,\rho ,m,\delta ,\gamma ) = \sum\limits_{j = 1}^{L} ( \tan (A_{j} ) - \tan (A_{j - 1} )) \cdot \\ \hfill SF(t,a_{j} ,b(\left| {A_{j} - A_{j - 1} } \right|,\rho_{j} ),m_{j} ,\delta_{j} ,\gamma_{j} ) \\ \end{aligned}$$

16 $$RCP(t,\alpha ,\theta ) = 1 - S\left( {\frac{t - \alpha }{\theta }} \right)$$

where *L* > 0 is the number of saccades of a sequence, *A*, *a*, *ρ*, *m*, *δ*, and *γ* are vectors whose components mean: *A*_*j*_ is movement angle in radians where $\pi / 2 > A_{j} > - \pi / 2$ and $A_{0} = 0,b( {| {A_{j} - A_{j-1}}|, \, \rho_{j} })$ determine saccadic slope, *ρ*_*j*_ is RFVS, *a*_*j*_ is the start time instant of a saccadic movement where $a_{j + 1} > a_{j} > 0$, *m*_*j*_ is decreased fixation slope, *δ*_*j*_ is the start time of decreased slope, *γ*_*j*_ is the duration; *α* and *θ* are real scalars which set the position of the returned saccade to the center localization of the eyeball and its slope. In (15) a tangent function was used because ±30° ocular movements are quasi linear [15].

Finally, EM(t) is obtained from a saccade sequence (15) and one single returned movement (16). Its expression is in (17)

17 $$EM(t) = \sum\limits_{i = 1}^{K} {RCP(t,\alpha_{i} ,b(\left| {A_{i,L(i)} } \right|,\tau_{i} } ) \cdot SS(t,A_{i} ,a_{i} ,\rho_{i} ,m_{i} ,\delta_{i} ,\gamma_{i} )$$

where *K* > 0 is the number of ocular movement sequences, *L*(*i*) > 0 is the number of saccades of sequence *i*, *A*_*i*_, *a*_*i*_, *ρ*_*i*_, *m*_*i*_, *δ*_*i*_, and *γ*_*i*_ are vectors of sequence *i* that were described in (15) and (16), *A*_*i*,*L*(*i*)_ is the last component of vector *A*_*i*_, *α*_*i*_ > *a*_*i*,*L*(*i*)_ is the localization of the returned saccade to the eyeball center and is larger than the last saccade component of vector *a*_*i*_, and *τ*_*i*_ is RFVS of this back eye movement.

### Sequence of overshoots

Overshoots can be defined as a Gauss function (18). This is an even-symmetry function, whose maximum value is 1; *μ* and $\sigma$ shift and contract/dilate it. Of its area, 99.99% is in the interval $[\mu - 4\sigma ,\mu + 4\sigma ]$, where its width is controlled through parameter $\sigma$. Thus, overshoot widths can be defined in this interval.

18 $$G\left( {t,\mu ,\sigma } \right) = e^{{ - 0.5 \cdot \left( {\frac{t - \mu }{\sigma }} \right)^{2} }}$$

A target localization error causes overshoots in the EOG signal, so they overlap with saccades [19, 20]. An overshoot sequence is defined in (19)

19 $$O(t) = \sum\limits_{i = 1}^{K} {\sum\limits_{j = 1}^{L(i)} {C_{i,j} \cdot G\left( {t,\mu_{i,j} ,\sigma_{i,j} } \right)} }$$

where *C*_*i*,*j*_ is the amplitude of overshoot, $\sigma_{i,j}$ is overshoot width (20), and, *μ*_*i*,*j*_ is the time localization of the overshoot that must be equal to (21); $b ( {|A_{i,j} - A_{i,j - 1} |, \, \rho_{i,j} })$ was defined in (10) and *θ*_*i*,*j*_ can be *a*_*i*,*j*_ if it is on saccade sequence or *α*_*i*_ if it is on returned saccade to center localization of the eyeball (17), and *sign*() is the sign function. In this way, overshoots range from 0.5 up to 0.999 in (9).

20 $$\sigma_{i,j} = \ln (999) \cdot \frac{{b\left( {\left| {A_{i,j} - A_{i,j - 1} } \right|,\rho_{i,j} } \right)}}{8}$$

21 $$\mu_{i,j} = \theta_{i,j} - 0.5 \cdot sign\left( {\frac{{\partial EM(\theta_{i,j} )}}{\partial t}} \right) \cdot b\left( {\left| {A_{i,j} - A_{i,j - 1} } \right|,\rho_{i,j} } \right) \cdot \ln \left( {\frac{1}{0.999} - 1} \right)$$

### Sequence of blinks

A blink has a higher slope before its peak than afterwards. So, this can be formed as (22).

*β*(*t*) is a piecewise function based on two Gauss functions, where one is double dilated in relation to the other. It is known that about 99% of the area of (18) is in the interval [*p* − 3*h*, *p* + 3*h*]. Thus, the interval [*p* − 3*h*, *p* + 6*h*] contains 99% of *β*(*t*) area.

22 $$\beta ( {t,p,h} ) = \left\{ {\begin{array}{ll}{G ( {t,p,h} )} & {t \le 0} \\{G( {t,p,2h})} & {t > 0} \\\end{array} } \right.$$

Equation (23) defines the blink signal where *R* is the number of blinks, *V*_*j*_ is the amplitude of one blink, *h*_*j*_ determines blink width, and *p*_*j*_ is the localization of the blink peak where $p_{j + 1} \ge p_{j} + 6h_{j}$.

23 $$B(t) = \sum\limits_{j = 1}^{R} {V_{j} \cdot \beta \left( {t,p_{j} ,h_{j} } \right)}$$

### Additive noise

Bioamplifiers are not perfect systems, and their outputs show interference from pink noise due to surface electrodes [36]. Therefore, we added this kind of noise, *n*(*t*), of 27 dB in relation to (17). This gave outputs which were more like real EOG signals from bioamplifiers.

## Appendix 2: Test settings

The model of “Appendix 1” was used in this paper’s tests, and its main parameters are summarized in Table 4.

**Table 4:** Test settings: main EOG-SG parameters

Pars.	Tests 1–3	Test 4	Test 5	Test 6	Test 7	Test 8
*K*	100	0	1,000	125	1	1,000
*L*	1	0	1	[6, 10]	1,000	1
*A* (º)	±40	0	±40	±15	±40	±40
*ρ*	[0.9, 1.1]	0	[0.9, 1.1]	[0.9, 1.1]	[0.9, 1.1]	[0.9, 1.1]
*m*	0	0	0	0	0	[0, 0.05]–[0.35, 0.40]
*TSS* (s)	[0.6, 1.5]	0	[0.3, 0.4]–[1.4, 1.5]	[0.3, 0.4]–[1.4, 1.5]	[0.3, 0.4]–[1.4, 1.5]	[0.6, 1.5]
*9h* (s)	[0.3, 0.55]	[0.1, 0.2]–[0.4, 0.5]	0	0	0	[0.3, 0.55]

Settings of tests. Square brackets mean random variations. Meaning of acronyms in the order of appearance.

*K* number of saccade sequences, *L* number of saccadic movements per sequence, *A* eye movement angle, *ρ* random factor of variability of saccade, *m* fixation slope, *TSS* time between two successive saccades, *9h* blink width, º degree, *s* second.

Three implicit concepts of EOG-SG must be described before explaining the signal generated in each test. The first is the time between sequences (TbS) in (17). This is defined as the period from the final saccadic movement (*α*_*i*_) of sequence *i* in (16) up to the first saccade *a*_*i*+*1*,*1*_ of the next sequence *i* + *1* in (15). Another concept is the rate of blinks in a minute (BM), which establishes a limit for the number of blinks. Therefore, this affects blink localizations (*p*_*j*_) in (23). However, if their positions are between sequences of saccade-fixation movements (17), as defined in (24), then blinks and ocular movements do not overlap. The last concept is when an overshoot happens. With these concepts defined, their values were set: TbS changed between 3 and 5 s for all tests (25); blinks happened between the sequence of ocular movements (24) and a frequency of 19 BM for Tests 1–3 and 5, between 12 and 22 BM for Test 4 and 0 BM in the others [21]; overshoots were generated when an eye shift exceeded +25° in absolute value from previous eye position in Tests 1–3 and 5. The others tests were without overshoots.

24 $$a_{i + 1,1} > p_{j} > \alpha_{i}$$

25 $$a_{i + 1,1} - \alpha_{i} \in [3,5]$$

The same setting was established for Tests 1–3, where parameters of proposal technique were obtained. They defined 100 sequences (*K*) of GBMs whose amplitudes (*A*) changed randomly between ±40°. This kind of movement occurs when the length of vector *A* is 1 in (17), that is, *L* is 1 in (15). The time between two successive saccades (TSS), that is, GBM width, was set between 0.6 and 1.5 s (26) with horizontal slope (*m* = 0) in (11). In contrast, blink width (9*h*) in (23) oscillated between 0.3 and 0.55 s [22].

26 $$a_{i,n + 1} - a_{i,n} \in [0.6,1.5]$$

Test 4 analyzed the ability to delete blinks. For this reason, the signals obtained were without saccade sequences (*K* = 0 and *L* = 0). Their widths were increased 0.1 s from an initial interval [0.1, 0.2] up to [0.4, 0.5] s.

Waveform preservation was analyzed in Tests 5–7. For this reason, the signals were free of blinks and overshoots. The distance between saccades changed 0.1 s in each iteration, [17], from an initial random value of [0.3, 0.4] up to [1.4, 1.5] s. The width limit of GBMs was studied in Test 5. 1,000 of them were generated with amplitudes between ±40°. Test 6 used signals with 125 sequences [7]. The total rank of eye movement was set in the interval [−15°, +15°]. The first saccade moved from the center position (0°) up to −15°, and the last shifted from +15° to center position (0°). Thus, the amplitude of stair-shaped waveforms was 30°. The number of saccades between them oscillated between 6 and 10. Test 7 was based on 1,000 random ocular movements (*L* = 1,000) with an amplitude between ±40° in a single sequence (*K* = 1), so that one saccade compared to the previous one could reach 80° of the distance.

The configuration of Test 8 was practically identical to Tests 1–3, but with 1,000 GBMs and non-horizontal fixation slopes, so they decreased 5% in each iteration, from [0, 5]% up to [35, 40]% of the slope.

Finally, the parameter of RFVS (*ρ*) in (10) was set between [0.9, 1.1] for all tests, so that the variability of the saccade slope was 20%.

## Abbreviations

EOG: electrooculogram
GBM: go-and-back movement
MF: median filter
FMH: FIR median hybrid filter
WFMH: weighted FIR median hybrid filter
CWFMH: center weighted FIR median hybrid filter
SWFMH: subfilter weighted FIR median hybrid filter
EFS: envelope filter sequence
EFS-WN: white noise signal generated by step 1 of EFS algorithm
SNR: signal–noise rate
FIFO: First In, First Out
IB: input-data buffer of online EFS
WNB: white-noise buffer of online EFS
OB: output buffer with the final fragment of online EFS output
CC: correlation coefficient
RMSE: root mean square error
EOG-SG: EOG system generation
SSV: saccadic slope variation
RFVS: random factor of variability of saccades
TbS: time between sequences
BM: blink in a minute
TSS: time between two successive saccades
